# Prefrontal hypoactivation induced via social stress is more strongly associated with state rumination than depressive symptomatology

**DOI:** 10.1038/s41598-023-41403-y

**Published:** 2023-09-13

**Authors:** Isabell Int-Veen, Andreas J. Fallgatter, Ann-Christine Ehlis, David Rosenbaum

**Affiliations:** 1 Department of Psychiatry and Psychotherapy, Tübingen Center for Mental Health (TüCMH), University Hospital of Tuebingen, Tübingen, Germany; 2 German Center for Mental Health (DZPG), Tübingen, Germany

**Keywords:** Psychology, Human behaviour

## Abstract

Previous studies have consistently shown a pattern of prefrontal hypoactivation in depressed patients (DP); however, it remains unclear whether this neural correlate is a consequence or concomitant feature of depression and/or whether ruminative thinking might be underlying. Using a sample comprising 65 healthy controls (HC) and 77 DP, we investigated the behavioral and neural correlates in response to stress and their association with depressive symptomatology, trait and state rumination. Fitting repeated-measurement MANOVAs including 21 fNIRS-channels covering the bilateral Inferior Frontal Gyrus (IFG), Dorsolateral Prefrontal Cortex (DLPFC) and Somatosensory Association Cortex (SAC), we investigated the predictive value of diagnostic group (HC vs. DP) and state rumination. In DP, we observed significantly lower increases in cortical oxygenation under stress in channels of the right IFG and bilateral DLPFC. Participants reporting lower state rumination and no increases in state rumination under stress showed higher increases in cortical oxygenation compared to the other groups and in more channels compared to the analysis on diagnostic group. Re-running our fNIRS-analysis while correcting for performance resulted in time-dependent changes dependent on group (DP vs. HC) no longer yielding significance, however for the differentiation of state rumination groups.

## Introduction

Rumination was initially defined as a cognitive vulnerability to develop depressive disorders and is therefore regarded as a trait construct that has been strongly associated with depressive psychopathology^1^. Trait rumination has been traditionally investigated using the Ruminative Response Scale (RRS), a subscale of the Response Style Questionnaire, which was originally developed by Nolen-Hoeksema and Morrow^2^. Approximately 15 years later, Robinson and Alloy^3^ discovered corresponding state rumination processes in a large community sample, which gave rise to the definition of trait and state aspects of ruminative thinking and the need for an appropriate distinction of them using questionnaires. In the same year, Treynor and Gonzalez^4^ further revised the original RRS due to substantial contentual overlap with symptoms of depression in order to assess ruminative thinking without depression-specific content. This was accompanied by the discussion of ruminative thinking as a cognitive vulnerability also being observable in the context of other psychopathologies^5–10^ which ultimately resulted in rumination being regarded as a transdiagnostic process^1^. From a neurobiological perspective, there are various findings on prefrontal hypoactivation in depressed patients (DP) (see e.g.^11–13^) using different experimental settings, methods and subtypes of depression (for a recent review see Pizzagalli and Roberts^14^). In short, especially the left Dorsolateral Prefrontal Cortex (DLPFC) has consistently been found to be hypo-activated during “affective and cognitive tasks requiring emotional or stress regulation, cognitive control, and/or shifting attention to external task demands”^14^. The authors point out that this might be due to reduced recruitment of the DLPFC in general and a cortical (PFC) inefficiency in DP. This means, most probably dependent on the type and need for resources, at some point healthy controls (HC) and DP might show similar DLPFC-activation but, with an increased need of resources, an aberrant functioning is observed on a neural and behavioral level.

Interestingly, in studies including experimental stress inductions by using for instance the Trier Social Stress Test (TSST)^15^, prefrontal hypoactivation was not only observed in DP^16^ but also in HC which were categorized as high trait ruminators according to the RRS^17, 18^. The TSST has not only been shown to be a very potent and ecologically valid stressor^19^ but also to be capable of eliciting stress-reactive rumination^16–23^. Consequently, high trait ruminators as well as DP showed higher increases in state rumination as induced via the TSST compared to low trait ruminators as well as HC^16, 17^.

There are only few studies investigating the neural correlates of state rumination, however first investigations also show aberrant DLPFC-functioning in HC during resting-state measurements^24^. Moreover, other prefrontal areas like the Medial Prefrontal Cortex, left Medial Orbito-Frontal Cortex but also several further regions like Precuneus and the Anterior Cingulate Cortex^25–31^ might be related to state rumination, but further research is needed to reach conclusive results. Please note that one issue in the literature of the neural correlates of state rumination involves the lack of a psychometrically evaluated and commonly used measure. Often, state rumination is assessed using the RRS which has been, as already mentioned, originally designed to assess trait rumination.

This is why we aimed to investigate the neural correlates of state rumination using a questionnaire specifically designed to assess state-processes in two previous studies of our lab^16, 32^. In both studies, DP and HC underwent the Trier Social Stress Test as well as two resting-state measurements before and after the stress induction while their cortical oxygenation was assessed using functional Near-Infrared Spectroscopy (fNIRS). We observed blunted prefrontal activation increases in DP compared to HC and overall reduced O2Hb-levels in the cognitive control network in DP; however analyzing our data using Regions of Interest (bilateral DLPFC, bilateral IFG and SAC), we were not able to perform post-hoc tests between different ROIs because of potential confounds of absolute differences due to different optical path lengths.

To the knowledge of the authors, no study has so far investigated the association of prefrontal hypoactivation with depressive symptomatology, trait and state rumination. While the well-known prefrontal hypoactivation for example under stress in DP is proposed to be associated with a reduced recruitment of the DLPFC in general and a cortical (PFC) inefficiency in DP, interestingly, prefrontal hypoactivation was not only observed in DP^16^ but also in HC which were categorized as high trait ruminators^17, 18^ and the influence of group membership on post-stress rumination was partly mediated by the reduced O2Hb-levels under stress in the left DLPFC^16^. This, with first preliminary results on the neural correlates of state rumination, gives rise to the question of whether trait measures (like the diagnostic group) or state measures (state rumination) might be two sides of the same coin and somewhat interchangeable. More specifically, in case depressive symptom severity, trait and state rumination are all not only highly intercorrelated on a psychological and behavioral basis but also associated with the same neural activation pattern, these findings would propose their interchangeability and implicate a discussion of shared and unique variance in a psychometric analysis.

In order to disentangle these interrelationships, we merged the samples of two previous functional Near-Infrared Spectroscopy (fNIRS) studies of our lab in order to increase the power to detect effects on a single-channel level^16, 32^. Like this, it is possible to assess a more fine-grained activation pattern which could inform future neuromodulation studies, which might then be used to investigate the aforementioned relationships by a direct modulation of DLPFC-activity (and thus unravel causal relations).

## Results

### State rumination

**Figure 1:**
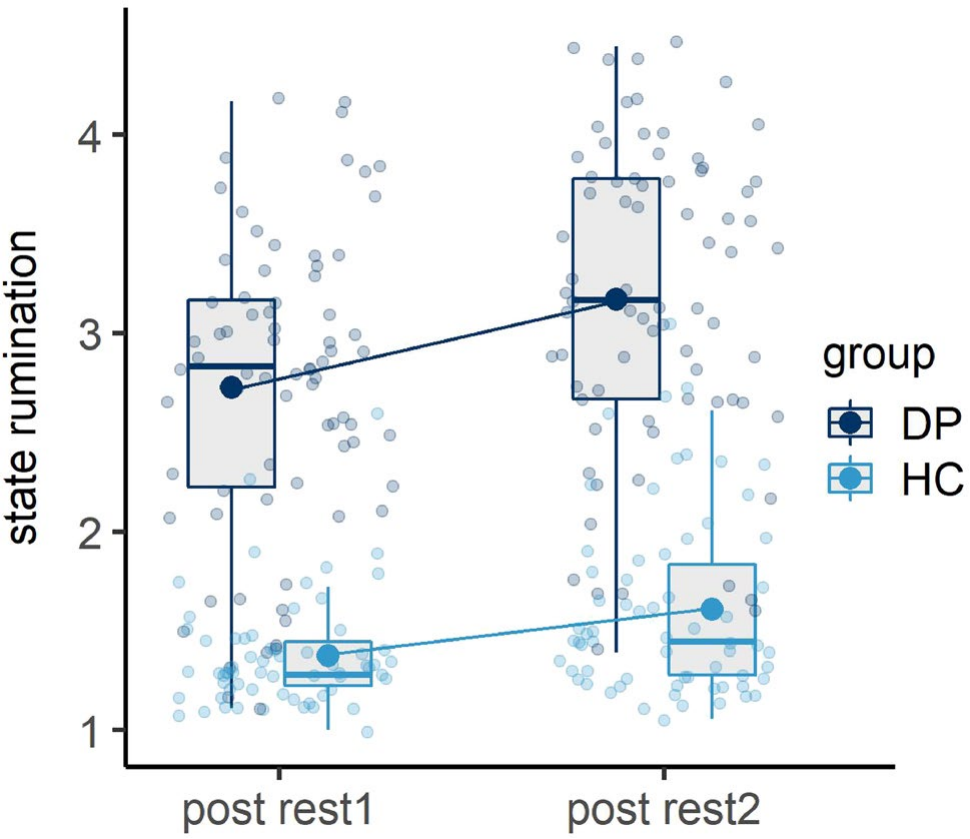
Line and boxplots of state rumination ratings. Transparent dots and boxplots indicate the raw data. The lower and upper hinges of the boxplot correspond to the first and third quartiles. Whiskers extend from the hinge to the largest value no further than 1.5 × Interquartile-Range. Bold dots and lines connecting the dots indicate the estimated marginal mean of the fitted models. *DP* depressed patients, *HC* healthy controls, *post rest1* assessment of state rumination after resting-state measurement 1 (baseline), *post rest2* assessment of state rumination after resting-state measurement 2 (post stress).

Investigating subjective state rumination ratings, we fitted a repeated measurements ANOVA (rmANOVA) dependent on time (post rest1 vs. post rest2) and group (DP vs. HC) and found a significant main effect of time, *F*(1, 138) = 46.163, *p* < 0.001, ${\eta }_{p}^{2}$ = 0.251, indicating an increase in state rumination due to the stress induction. We further observed a significant main effect of group, *F*(1, 138) = 250.633, *p* < 0.001, ${\eta }_{p}^{2}$ = 0.645, reflecting higher state rumination in general in the case of DP compared to HC, as well as a significant interaction of time and group, *F*(1, 138) = 4.388, *p* < 0.05, ${\eta }_{p}^{2}$ = 0.031, reflecting higher increases in the case of DP (see Fig. 1).

### Performance under stress

**Figure 2:**
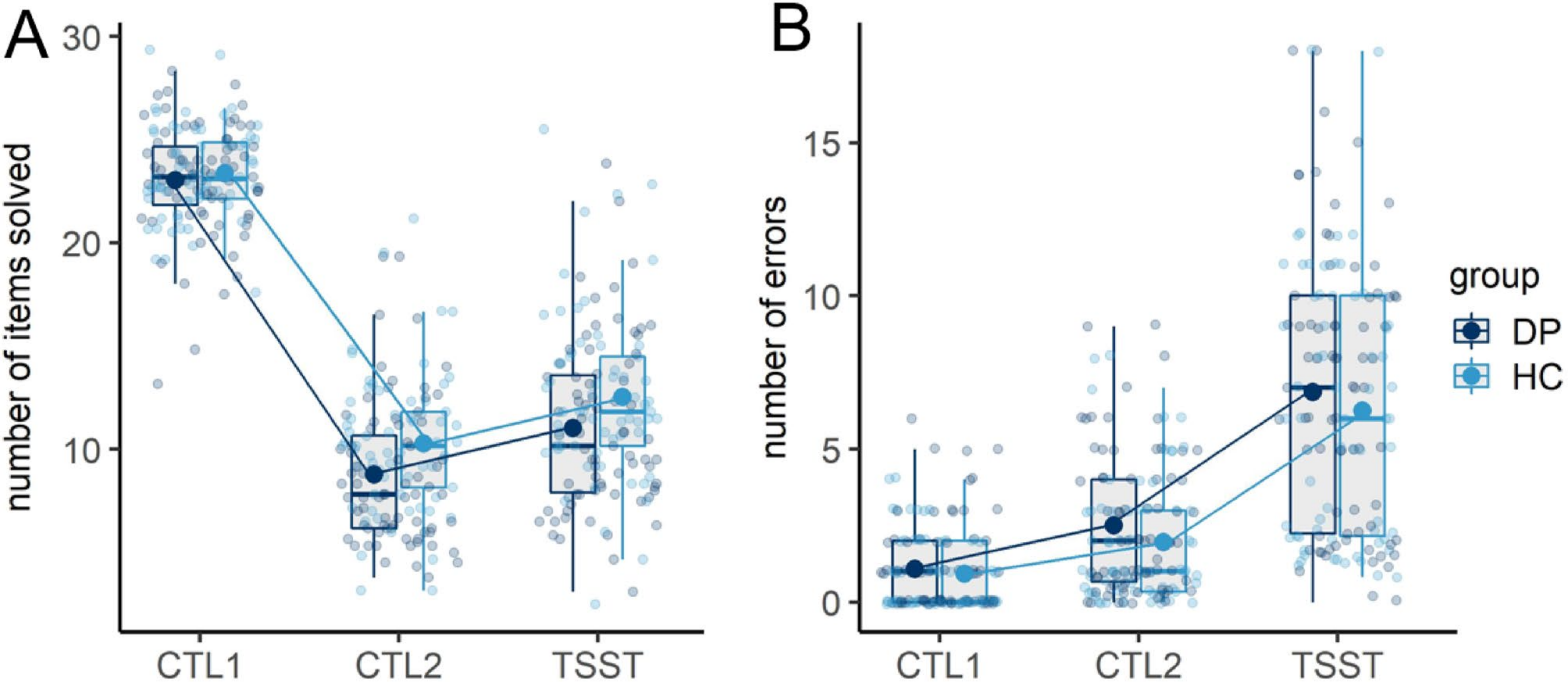
Line and boxplots of the mean number of items solved (**A**) and total number of errors (**B**) dependent on group (DP vs. HC). Transparent dots indicate the raw data. Bold dots and lines connecting the dots indicate the estimated marginal mean of the fitted models. The lower and upper hinges of the boxplot correspond to the first and third quartiles. Whiskers extend from the hinge to the largest value no further than 1.5 × Interquartile-Range. *DP* depressed patients, *HC* healthy controls, *CTL1* control task 1 (reading numbers aloud), *CTL2* control task 2 (performing calculations without social stress), *TSST* arithmetic task of the TSST (performing calculations under social stress).

Investigating the performance of mental arithmetics, we fitted a rmANOVA on the mean number of items solved dependent on group (DP vs. HC) and time (control task 1 (CTL1) vs. control task 2 (CTL2) vs. arithmetic task of the TSST). We observed a significant main effect of time, *F*(1.252, 171.549) = 992.468, *p* < 0.001, ${\eta }_{p}^{2}$ = 0.879. In general, HC solved on average 1.5 more items compared to DP, which was reflected by a significant main effect of group, *F*(1, 137) = 6.148, *p* < 0.05, ${\eta }_{p}^{2}$ = 0.043. Post-hoc tests of the main effect of time revealed that participants performed significantly (*p* < 0.05) less calculations (CTL2 and TSST) compared to reading numbers (CTL1) and in case they were instructed to calculate as fast and as correctly as possible (TSST) significantly more compared to CTL2. When analyzing the number of errors, we did not observe any differences dependent on group but again a significant main effect of time, *F*(1.442, 197.593) = 167.689, *p* < 0.001, ${\eta }_{p}^{2}$ = 0.550. Pairwise comparisons indicated significantly more errors during CTL2 as well as the TSST compared to CTL1 and during the TSST compared to CTL2 (see Fig. 2).

Fitting the same rmANOVA dependent on the state rumination cluster variable (SR-cluster, see methods section), we observed a significant main effect of time, *F*(1.258, 169.852) = 910.201, *p* < 0.001, ${\eta }_{p}^{2}$ = 0.871, a significant main effect of SR-cluster, *F*(3, 135) = 6.669, *p* < 0.001, ${\eta }_{p}^{2}$ = 0.129, as well as a significant interaction of time and SR-cluster, *F*(3.774, 169.852) = 2.507, *p* < 0.05, ${\eta }_{p}^{2}$ = 0.053. Benjamini–Hochberg-corrected post-hoc tests of the interaction of time and SR-cluster indicated an overall significantly (*p* < 0.05) higher number of solved items in the case of cluster 4 (low state rumination and little to no increase in state rumination due to the stress induction) when compared to cluster 3 (low state rumination and increase in state rumination due to the stress induction) and cluster 1 (high state rumination and increase in state rumination due to the stress induction) whereas cluster 4 and 3 only differed during the arithmetic task of the TSST and cluster 4 and 1 differed at CTL2 as well as the arithmetic task of the TSST.

**Figure 3:**
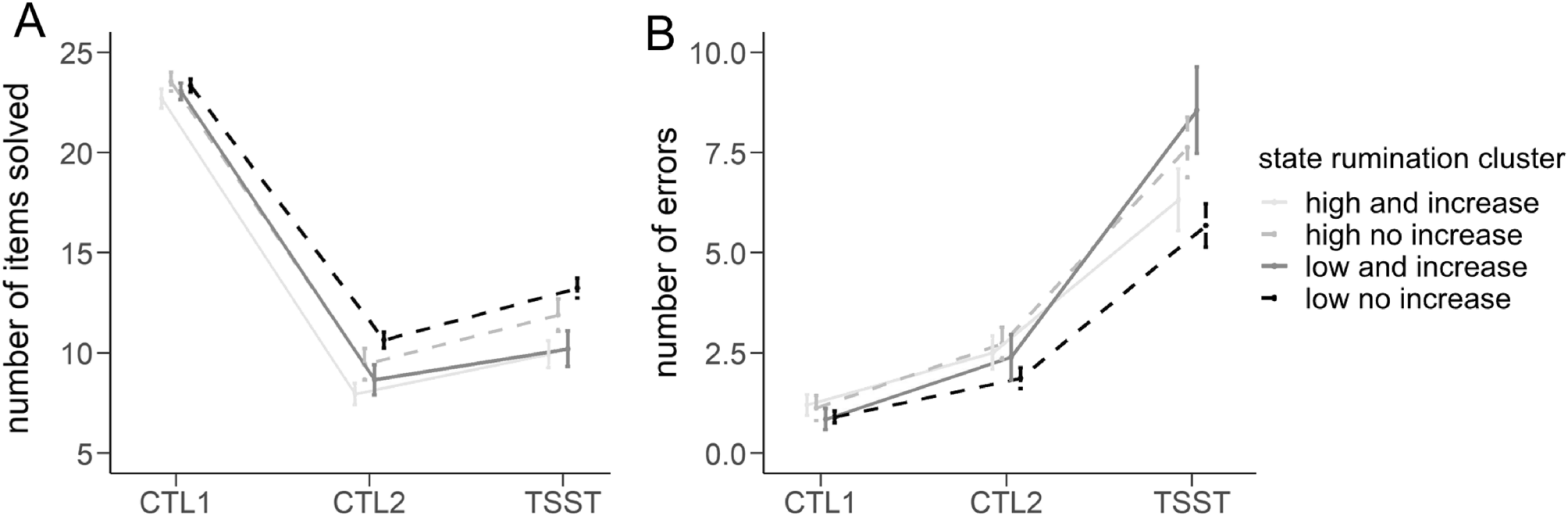
Line graphs of the mean number of items solved (**A**) and total number of errors (**B**) dependent on the state rumination cluster. *CTL1* control task 1 (reading numbers), *CTL2* control task 2 (performing calculations without social stress), *TSST* arithmetic task of the TSST (performing calculations under social stress).

Concerning the number of errors, we observed a significant main effect of time, *F*(1.461, 197.249) = 173.345, *p* < 0.001, ${\eta }_{p}^{2}$ = 0.562, as well as a significant interaction effect of SR-cluster and time, *F*(4.383, 197.249) = 2.373, *p* < 0.05, ${\eta }_{p}^{2}$ = 0.050. Benjamini–Hochberg-corrected pairwise comparisons revealed significant increases in errors over time (CTL1 vs. CTL2 vs. TSST). Investigating the interaction effect of time and SR-cluster, Benjamini–Hochberg-corrected post-hoc tests indicated no significant differences between the 4 groups during CTL2 nor during the TSST (uncorrected pairwise comparisons indicated a significant difference between cluster 3 and 4 in the case of the arithmetic task of the TSST) but significant increases (*p* < 0.05) in each of the 4 groups from CTL1 to CTL2 to the TSST (see Fig. 3).

### Neural correlates

Next, we fitted a rmMANOVA dependent on time (control task 1 (CTL1), control task 2 (CTL2) and arithmetic task of the TSST) and group (DP vs. HC). This rmMANOVA resulted in a significant main effect of time, *F*(42, 512) = 2.987, *p* < 0.001, *wilks λ* = 0.645, ${\eta }_{p}^{2}$ = 0.197, as well as a significant interaction of time and group, *F*(42, 512) = 1.681, *p* < 0.01, *wilks λ* = 0.772, ${\eta }_{p}^{2}$ = 0.121.

We firstly investigated the univariate tests of the main effect of time. Consequently, time yielded a significant predictor (*p* < 0.05) in all channels except for channel 19 and 21 (both right IFG).

The interaction of time and group yielded significance in the case of two out of three channels of the left DLPFC (channel 11, 12), two out of three channels of the right DLPFC (channel 20, 23), one out of three channels of the right IFG (channel 21) as well as two out of nine channels of the SAC (channel 35, 36). Investigating the pairwise comparisons of the interaction of time and group, we observed significant differences between HC and DP during CTL2 in the case of channel 20 (right DLPFC) and significant differences between HC and DP during the arithmetic task of the TSST in the case of two channels of the left DLPFC (channel 11, 12), one channel of the right IFG (channel 21) and one channel of the right DLPFC (channel 23). We further observed significant increases between CTL1 and the TSST—but only in HC—in the case of all the aforementioned IFG and DLPFC channels (channel 11, 12, 21, 23), two channels of the SAC (channel 35, 36) and another channel of the right DLPFC (channel 20). A subgroup of those further showed significant increases from CTL2 to TSST in the case of HC (channel 11, 12, 21, 36) and in the case of DP one channel of the right DLPFC (channel 20) (see Table 1). For an illustrative comparison of cortical oxygenation dependent on channel and group (DP vs. HC) see Supplementary Material S4.

**Table 1:** Illustration of the significant Benjamini–Hochberg-corrected time by group interactions (depicted as +) of the repeated measurements MANOVAs investigating fNIRS cortical oxygenation (group = depressed patients (DP) vs. healthy controls (HC), *SR-cluster* state rumination cluster).

ROI	Left IFG			Left DLPFC			Right IFG			Right DLPFC			SAC								
Channel	6	7	9	10	11	12	18	19	21	20	23	24	25	26	27	28	30	31	32	35	36
Group					+	+			+	+	+									+	+
SR-cluster	+			+	+	+			+	+		+		+							+

*IFG* Inferior Frontal Gyrus, *DLPFC* Dorsolateral Prefrontal Cortex, *SAC* Somatosensory Association Cortex.

Lastly, we fitted a rmMANOVA dependent on time and state rumination cluster and observed a significant main effect of time, *F*(42, 504) = 2.395, *p* < 0.001, *wilks λ* = 0.695, ${\eta }_{p}^{2}$ = 0.166, and a significant interaction effect of time and state rumination cluster, *F*(126, 1468.91) = 1.437, *p* < 0.01, *wilks λ* = 0.510, ${\eta }_{p}^{2}$ = 0.106.

Univariate tests yielded a significant (*p* < 0.05) main effect of time in one channel of the right DLPFC (channel 20), two channels of the left IFG (channel 7, 9) and two SAC-channels (channel 25, 32).

**Figure 4:**
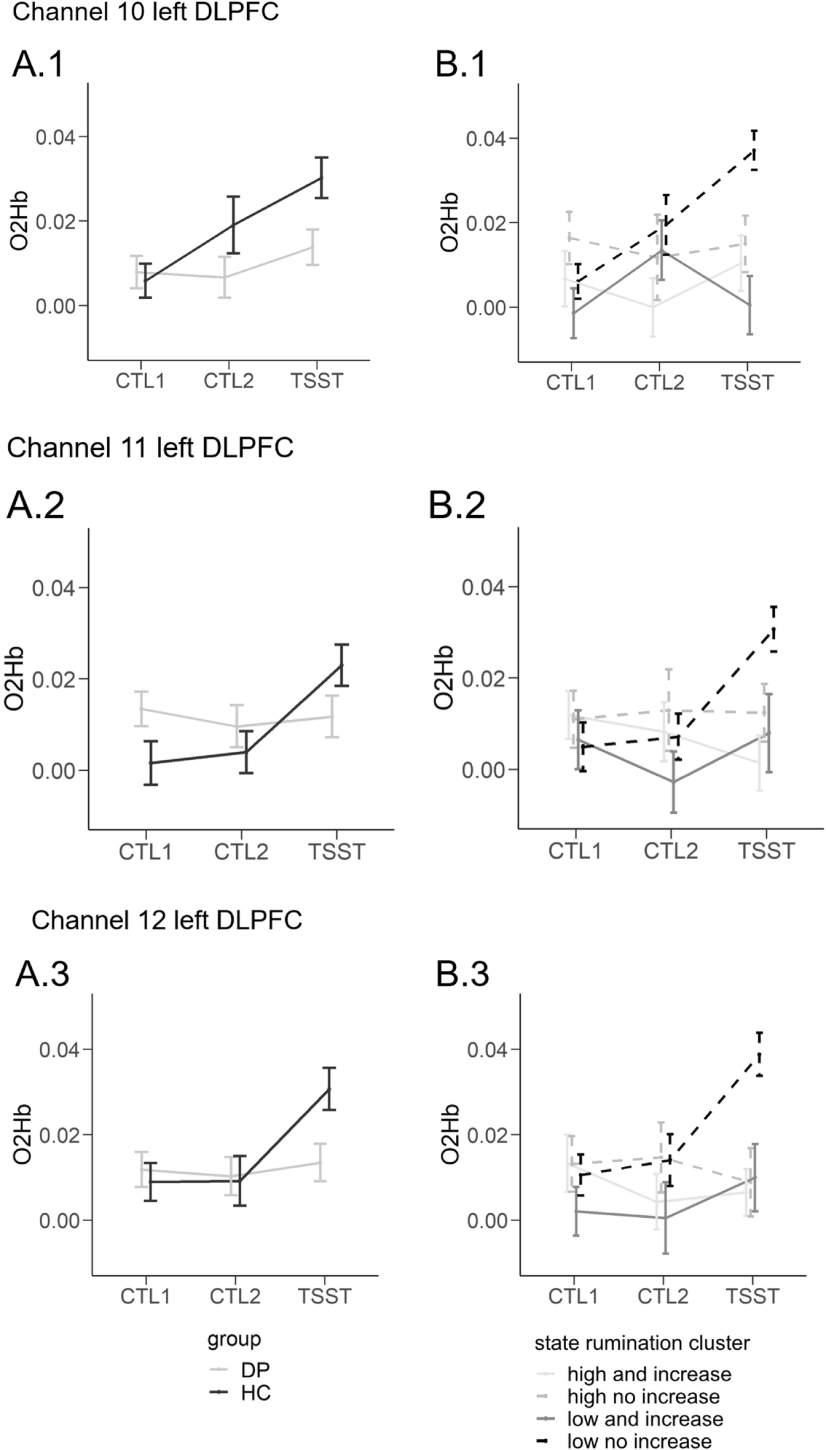
Illustration of the results of the repeated measurements fNIRS-MANOVAs for each channel of the left DLPFC and group (**A** = depressed patients (DP) vs. healthy controls (HC); **B** = state rumination cluster). For an illustration of the probeset where the channels are exactly located, we refer to Supplementary Material S2. *CTL1* control task 1 (reading numbers), *CTL2* control task 2 (performing calculations without social stress), *TSST* arithmetic task of the TSST (performing calculations under social stress). Please note that the interaction effect of time and group was significant in all channels of the left DLPFC besides channel 10 in the case of DP vs. HC. We depicted it either way for completeness. Error bars indicate ± 1 SE.

**Figure 5:**
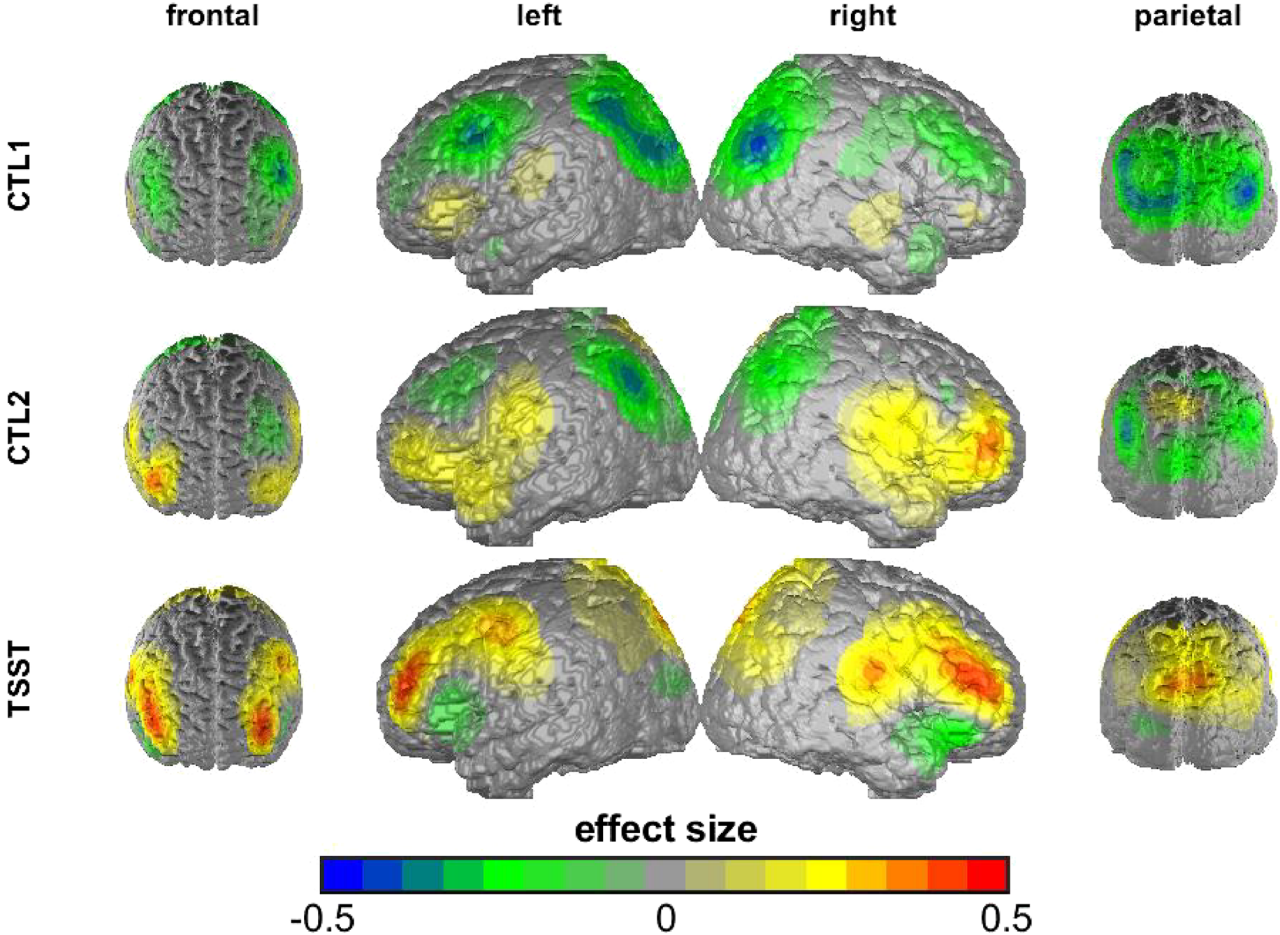
Differences in O2Hb-levels (not corrected for performance) dependent on group (DP vs. HC) during control task 1 (CTL1 = reading numbers), control task 2 (CTL2 = performing calculations without social stress) and TSST (performing calculations under social stress). Warm colors indicate higher O2Hb-levels in the HC compared to the DP; cool colors vice versa. Differences are depicted in Cohen’s *d*.

Univariate tests of the time by SR-cluster interaction yielded significance in the case of all three channels of the left DLPFC (channel 10, 11, 12), two channels of the right DLPFC (channel 20, 24), one channel out of three of the left (channel 6) and right IFG (channel 21) as well as two out of the nine SAC-channels (channel 26 and 36) (for a summary of the channels in which the interaction of time and SR-cluster yielded significance in the univariate tests, please see Table 1). We investigated the pairwise comparisons of the significant interaction of time and SR-cluster and observed significant differences at CTL2 between cluster 1 and 4 in the case of channel 20. Further, significant differences during the arithmetic task of the TSST have been observed between cluster 1 (high state rumination and increase) and 4 (low state rumination and little to no increase) in all channels covering the left DLPFC, one channel of the right IFG (channel 21), two channels of the right DLPFC (channel 20, 24) and two out of nine channels of the SAC (channel 26, 36). Significant differences during the arithmetic task of the TSST were observed between cluster 2 (high state rumination with little to no increase) and cluster 4 (low state rumination and little to no increase) in the case of three channels (one left DLPFC (channel 12), one right DLPFC (channel 24), one SAC (channel 36)). Significant differences during the arithmetic task of the TSST were also found between cluster 3 (low state rumination and increase) and 4 (low state rumination and little to no increase) in the case of six channels (two left DLPFC (channel 10, 12), two right DLPFC (channel 20, 24), one right IFG (channel 21), one SAC (channel 26)). With respect to changes between the different tasks, only cluster 4 (low state rumination and little to no increase) showed significant increases in cortical oxygenation between CTL1 and TSST in the case of all channels of the left DLPFC, one channel of the left IFG (channel 6), two channels of the right DLPFC (channel 20, 24), one channel of the right IFG (channel 21) and two channels of the SAC (channel 26, 36). Increases between CTL2 and the TSST were observable in all of the aforementioned channels but one channel of the right DLPFC (channel 24). For an illustration of the previously reported effects, see Figs. 4 and 5.

### Performance-corrected fNIRS-data

In order to account for the differences in performance in calculations during the TSST arithmetic task, we fitted a rmMANOVA dependent on time (control task 1, control task 2 and arithmetic task of the TSST) and group (DP vs. HC) using our fNIRS-data corrected for the number of items solved. This rmMANOVA again resulted in a significant main effect of time, *F*(42, 508) = 4.525, *p* < 0.001, *wilks λ* = 0.530, ${\eta }_{p}^{2}$ = 0.22; however, the interaction effect of time and group did no longer yield significance, *F*(42, 508) = 1.245, *p* = 0.145, *wilks λ* = 0.822, ${\eta }_{p}^{2}$ = 0.093. Univariate ANOVAs yielded time to be a significant predictor (*p* < 0.05) in the case of all channels except for channel 7, 18 and 21.

Fitting the same rmMANOVA using the corrected fNIRS-data dependent on time and state rumination cluster, we observed similar results as without correcting for performance, namely a significant main effect of time, *F*(42, 500) = 4.225, *p* < 0.001, *wilks λ* = 0.545, ${\eta }_{p}^{2}$ = 0.262, and a significant interaction of time and SR-cluster, *F*(126, 1457.309) = 1.245, *p* < 0.05, *wilks λ* = 0.530, ${\eta }_{p}^{2}$ = 0.094. Investigating the univariate tests, we found time to yield a significant predictor in the case of all channels of the right DLPFC, one channel of the left DLPFC (channel 10), seven out of nine channels of the SAC (channel 25, 26, 27, 28, 30, 32, 35) as well as one channel of the left (channel 6) and right IFG (channel 19), respectively.

**Figure 6:**
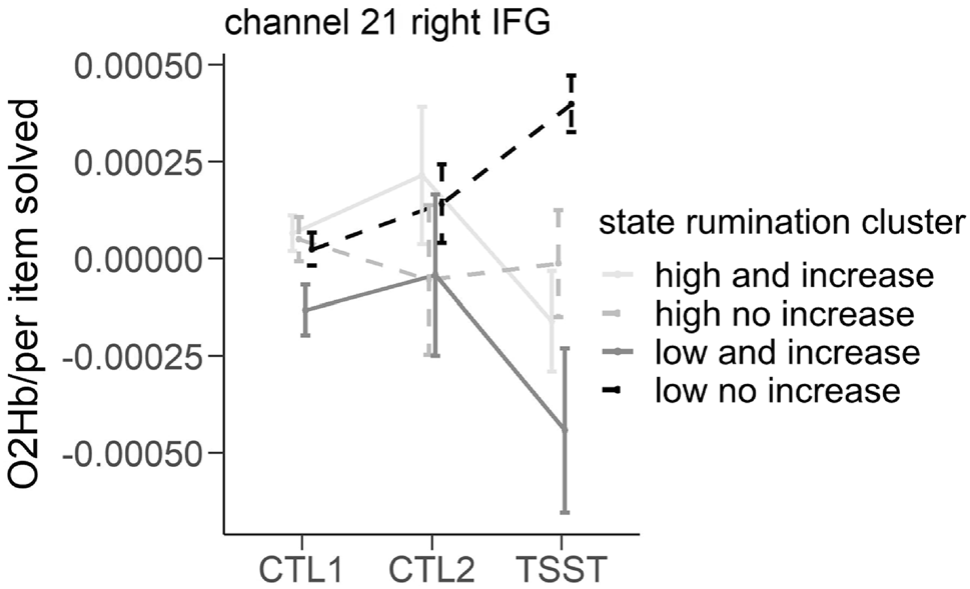
Illustration of the results of the repeated measures fNIRS-MANOVA using the performance-corrected fNIRS data in the case of channel 21 dependent on state rumination cluster during control task 1 (CTL1 = reading numbers), control task 2 (CTL2 = performing calculations without social stress) and TSST (performing calculations under social stress).

Investigating the significant interaction of time and SR-cluster, univariate tests revealed that it was a significant predictor only in channel 21 (right IFG). Pairwise comparisons revealed significant increases in cortical oxygenation in channel 21 from CTL1 to the TSST only in the case of cluster 4 (low state rumination and little to no increase in state rumination due to the stress induction). During the TSST, cluster 4 (low state rumination and little to no increase) showed significantly higher cortical oxygenation compared to cluster 3 (low state rumination and increase) and cluster 1 (high state rumination and increase). For an illustration see Fig. 6.

## Discussion

Prefrontal hypoactivation is an intensely studied phenomenon in the context of depression. With the use of several neuroimaging techniques, this neural correlate has been repetitively replicated and is now regarded to be a robust finding across different samples (for a review see Pizzagalli and Roberts^14^). As a major vulnerability to develop depressive episodes, rumination has also been found to be linked to prefrontal hypoactivation. Interestingly, however, rumination is not only apparent in DP but also in HC and first studies on state rumination also show aberrant DLPFC-functioning in HC^24^. Due to a rather scarce literature, it remains unclear whether the aforementioned neural correlates might be a concomitant feature of symptoms of depression, or whether trait or state rumination might (also) account for this characteristic neural activation pattern. As ruminative thinking is assumed to be a transdiagnostic factor and part of the psychopathological abnormalities in various disorders^5–9^, this might have crucial implications for a more coherent model of neural pathways assumed to be involved in these disorders and consequently also in the respective treatment.

To investigate these associations, we merged the samples of two of our previous studies on rumination investigating the cortical hemodynamic changes using fNIRS and behavioral alterations of HC and DP when stress is induced using the TSST. Merging these two studies with an equivalent experimental set-up resulted in a total sample of N = 142, which increased the power to detect even smaller effect sizes. Intuitively, high levels of trait rumination are nearly in all cases simultaneously present with increased symptoms of depression and therefore more often apparent in DP. These issues of multicollinearity do pose major problems in the differentiation of the effects which is why we only investigated “trait measures” (BDI-II, RRS and diagnostic group (DP vs. HC)) and state rumination in separate models. Statistical models including corresponding other variables as covariates would result in interpretations of the predictor “in case the other variable is held constant”. This, in fact, would pose a rather artificial situation and potentially lead to biased estimates, which is why we opted for the aforementioned analysis. We are, as a consequence, not able to estimate the amount of shared and unique variance of the trait variables as well as state and trait measures, nor estimate whether one model might be a significantly better fit for the data; however, we are able to compare trait and state aspects in their predictive value of behavioral and neural responses due to the stress induction.

We firstly investigated the behavioral stress response including state rumination and performance in control tasks and the TSST. Intuitively and as shown in the previous analysis of the subsamples of this data, DP showed higher increases in state rumination after social stress compared to HC, and the latter further performed generally better. Better performance was reflected by more items solved in the control tasks and the TSST, whereas we did not observe less errors in HC compared to DP. In order to investigate the effect of state rumination while considering change scores and post-stress state rumination, we combined both in one variable: In SPSS, we used hierarchical clustering (average linkage between-groups) and extracted 2–5 clusters and according to the dendrogram, four “state rumination clusters” emerged: One group of participants showing generally high levels of state rumination and increases due to the stress induction (cluster 1), one group with high levels of state rumination but little to no increase (cluster 2), one group with generally low levels of state rumination and increases due to stress (cluster 3) as well as a group with low state rumination with little to no increases (cluster 4). Please note that cluster 4 primarily comprised HC, cluster 1 and 2 comprised primarily DP and cluster 3 both. This differentiation on a behavioral level was of major interest concerning potentially distinct neural response patterns to stress. That is, as state rumination is apparent in HC and DP, we were interested in whether prefrontal hypoactivation in distinct channels would be associated with only, for instance, both clusters with state rumination increases which would question the more general categorization of HC vs. DP in case of the investigation of prefrontal hypoactivation.

Concerning the performance during the TSST, state rumination clusters reflected more or less the same but more fine-grained results as the analysis of “trait”-group (DP vs. HC): We observed cluster 4 (low state rumination and little to no increase ~ HC) to perform better compared to cluster 3 (low state rumination and increase) as well as cluster 1 (high state rumination and increase). That means, participants showing increases in state rumination due to the stress induction performed worse compared to groups with little to no increase. This is well in line with the idea of a less efficient recruitment of the prefrontal areas plus cortical inefficiency in DP in the case of “affective and cognitive tasks requiring emotional or stress regulation, cognitive control, and/or shifting attention to external task demands”^14^, which is ultimately reflected by a worse performance on a behavioral level. However, on the other hand, this might also be a concomitant feature of lower motivation and other factors inducing malperformance as a recent meta-analysis found that worse performance of depressed patients in neuropsychological tests might be overestimated^33, 34^ and it is reasonable to assume that the neuronal correlates could then also be exaggerated. However, to the knowledge of the authors, there is no analogous investigation concerning neural data of depressed patients, so far. When analyzing fNIRS-data dependent on group (DP vs. HC) using a repeated measures MANOVA, we observed significant increases in cortical oxygenation in nearly all channels due to the stress induction as well as time-dependent differences between HC and DP: In DP we observed prefrontal hypoactivation under stress in the case of two out of three channels of the left and right DLPFC, respectively, as well as one channel of the right IFG. Following the extensive review concerning prefrontal cortex alterations in depression by Pizzagalli and Roberts^14^, especially the results of aberrant functioning in the left DLPFC being associated with depression are well in line with an extensive body of literature. While there is a clear focus on the left DLPFC in the literature, recent meta-analyses of randomized controlled trials using repetitive transcranial magnetic stimulation over the right DLPFC also evaluated them as successful in the treatment of DP^35, 36^. Future studies are needed to evaluate and integrate these findings and extend the literature on the exact underlying neural pathways.

When we investigated the neural correlates dependent on state rumination clusters, we firstly found nearly the same channels being associated with time-dependent changes as in our previous analyses using “trait”-groups, but crucially more channels: For instance, we now observed significantly lower activation in all channels of the left DLPFC in the case of cluster 1 (high state rumination and increase) when compared to cluster 4 (low state rumination and little to no increase  ~ HC). This translates to a specific sub-group of DP showing a prefrontal activation pattern significantly differing from HC, which is identified through their respective pattern of state-rumination-reactivity. This finding suggests that the mere differentiation of diagnostic groups (clinical vs. non-clinical, i.e. meeting a predefined number of symptom categories or not) might not comprehensively explain state-dependent brain activity. Again, due to problems of multicollinearity and the current sample size, we are not able to investigate shared and unique variance to trait or state measures; however, this data suggests this might be an interesting endeavor. This is even clearer when we recap the results of the same rmMANOVA using performance-corrected fNIRS-data. As previously reported, we observed significant differences of both, DP vs. HC as well as the different SR-clusters, in the performance during (the non-stressful control tasks and) the arithmetic task of the TSST. In this rmMANOVA, the interaction-effect of time and group (DP vs. HC) and the corresponding prefrontal hypoactivation in DP vanished in the case of our “trait”-analysis. This was, however, not the case for SR-cluster. Here, the interaction effect of time and SR-cluster remained significant. Using Benjamini–Hochberg-corrected post-hoc tests, we observed significant increases in cortical oxygenation in channel 21 (right IFG) from CTL1 to the TSST only in the case of cluster 4 (low state rumination and little to no increase). Please note that this was the result after correction of multiple testing as we analyzed a total of 21 channels to be able to tell where exactly differences were present. Most probably, a larger sample size with balanced groups would be beneficial in evaluating this effect. Generally speaking, however, state measures do pose an important predictor when investigating stress-induced prefrontal hypoactivation and future studies should consider state measures in group differentiation rather than—or in addition to—trait measures.

One limitation concerning the interpretation of the aforementioned results concerns the penetration depth of fNIRS, which is estimated to 3 cm. While the cortex can be captured, deeper brain structures cannot be assessed. In this case, further studies using other neuroimaging techniques are needed to investigate the neural correlates of state rumination as regions like the Anterior Cingulate Cortex are also discussed to be involved^25, 26^. A combined fMRI- and fNIRS-study using neuro-navigation software would be able to quantify between-subjects as well as within-subjects (over the course of the experiment) variability of channel placement and also assess deeper underlying brain regions. Nevertheless, in order to ensure high ecological validity, the merged studies used fNIRS as it is less prone to motion artifacts^37^ and participants are able to stand in front of the jury members as in the original TSST^15^.

The findings presented in our analyses add to a very scarce literature on the neural correlates of ruminative processes as well as the differentiation of state and trait aspects in these neural aberrations. Aforementioned results might be used in experimental studies investigating the causal link between neural activity and rumination using neuromodulation to gain insights into the underlying mechanisms. Repetitive transcranial magnetic stimulation (rTMS) for instance has been proposed as a noninvasive approach to alter brain excitability^38^. It is nowadays regarded as a useful adjunct to the treatment of depression complementary to antidepressant medication and psychotherapy. rTMS, and especially Theta Burst Stimulation (TBS) has been found to result in longer-lasting effects, which were already found to be effective in the treatment of depression^39–42^; however, the exact underlying mechanism and especially the interplay of depressive symptoms and rumination is so far not well understood. One pioneer study by De Witte et al.^43^ applied intermittent (i.e., facilitating) TBS (iTBS) to the left DLPFC and observed a—although only marginally significant—buffering effect of iTBS on increases in state rumination in high trait ruminators. Following their idea, we recently conducted a study using continuous (i.e., inhibiting) TBS (cTBS) and iTBS in a sample of low and high trait ruminators^44^. After investigating behavioral effects on state rumination, we also plan on conducting a TBS-fNIRS-study in order to assess the direct neural correlates of the stimulation. In the long run, these findings might lead to insights that could form the basis for better treatments of depression and other mental disorders associated with rumination.

## Methods

### Recruitment

Participants merged in this analysis were originally recruited within two distinct studies, which however followed the same recruitment procedure. In the first study we recruited 23 HC and 22 DP, in the second study 42 HC and 55 DP. Exclusion criteria for both studies were any disorder or medical condition affecting the cerebral metabolism, heart rate variability and/or cortisol levels: Diabetes mellitus, kidney insufficiency, hypertension, dysrhythmia, Cushing syndrome, substance abuse, adrenal insufficiency, cortisone medication, pacemaker, craniocerebral trauma as well as any medication except for oral contraceptives (or antidepressants in case of DP). HC were excluded in case they had any acute mental disorder and were additionally screened prior to study inclusion using the Structured Clinical Interview (SCID)^45^ by trained psychologists. For DP, furthermore any other primary mental disorder except ICD-10 diagnosis F32.x, F34.1 and F33.x was excluded in addition to subjects with acute suicidal tendencies, extraordinarily severe depressive symptoms (BDI-II > 50), deficient emotional stability according to the currently treating psychologist and decompensation under social stress in the past. HC were recruited via circular emails; DP were recruited at the University Hospital of Tübingen and via ambulant psychotherapists. All procedures were approved by the ethics committee at the University Hospital and University of Tübingen and are in line with the Declaration of Helsinki in its latest version. All participants gave their written informed consent prior to data collection.

### Procedures

**Figure 7:**
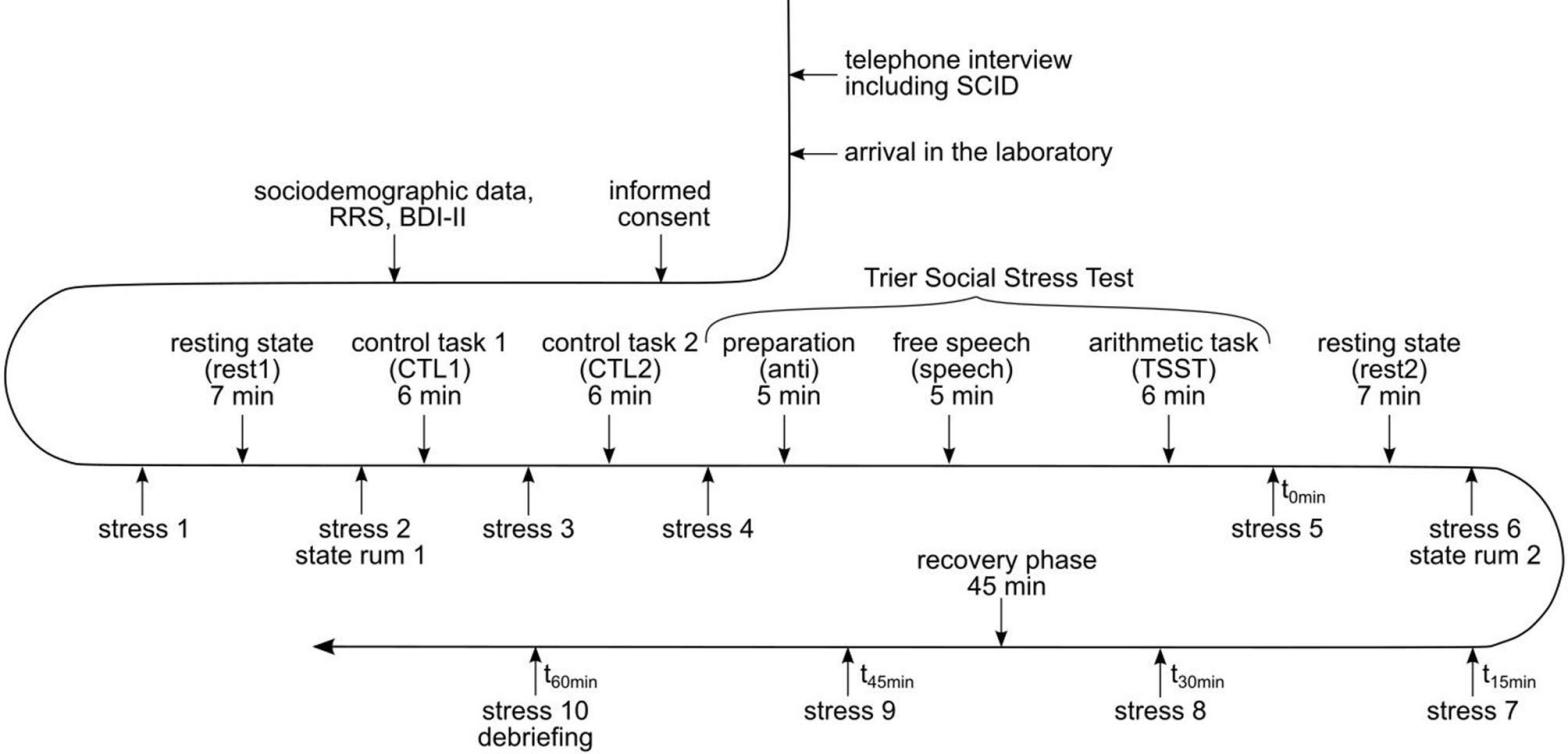
Time course of both studies. *SCID* Structured Clinical Interview, *RRS* Ruminative Response Scale, *BDI-II* Beck Depression Inventory II, *stress* Visual Analogue Scale assessing subjective stress (0–100%).

For both studies the experimental procedure was the same (see Fig. 7). At first, baseline questionnaires assessing demographic data, depressive symptoms (Beck Depression Inventory II; BDI-II; German version by Hautzinger et al.^46^) and trait rumination (Ruminative Response Scale; RRS^2^) were assessed. Meanwhile, participants were prepared for the fNIRS-measurement which was assessed pre-stress, during the stress induction and for 7 min post-stress. After the administration of the questionnaires, a 7 min resting-state measurement (rest1) was performed where participants were instructed to sit still while letting their mind wander and keeping their eyes open. Following this, two control tasks were performed: For each of the 6 trials of control task 1 (CTL1), participants were given number sequences they had to read out aloud for 40 s which was followed by 20 s rest allowing a recovery of the hemodynamic response. In case they made an error, those were not pointed out and participants did not have to start all over again. In the end, the number of numbers read out aloud and errors per trial were documented. For each of the 6 trials of control task 2 (CTL2), participants were given different numbers from which they had to sequentially subtract 13 for 40 s, followed by 20 s rest. During the control tasks, only a friendly study nurse operating the fNIRS-device was present and participants were instructed to complete the tasks at their own pace. In case they made an error in CTL2, participants had to start all over again from the respective starting point. Afterwards, the number of errors made and the number of calculations per trial were documented. After both control tasks, two experimenters who remained socially non-responsive and were wearing white coats entered the room for the stress-induction using the Trier Social Stress Test (TSST)^15^. Participants were instructed to imagine having applied for a job at the University Hospital and part of the job interview was to give a speech about their personal strengths and qualifications. During a 5 min anticipation phase, participants had time to prepare themselves before the experimenters took away their notes, instructed them to stand up and deliver the speech. After 5 min, the experimenters instructed an arithmetic task analogue to CTL2, but this time participants had to calculate as fast and as correctly as possible while holding eye-contact with one of the experimenters. The other experimenter documented the number of performed calculations and errors. Afterwards, the experimenters left the room without any comment and a second resting-state analogue to the first one was conducted. Throughout the experiment, participants rated their subjective stress using Visual Analogue Scales (0–100%) on one page so they could allow for their last rating. After both resting-states, state rumination was assessed using a questionnaire that was already evaluated in other studies (Int-Veen et al., in preparation; for the items, see Supplementary Material S1). Lastly, a post-stress phase of 45 min resting followed in which subjective stress was assessed every 15 min (for further details see^32^).

### Neural correlates

**Figure 8:**
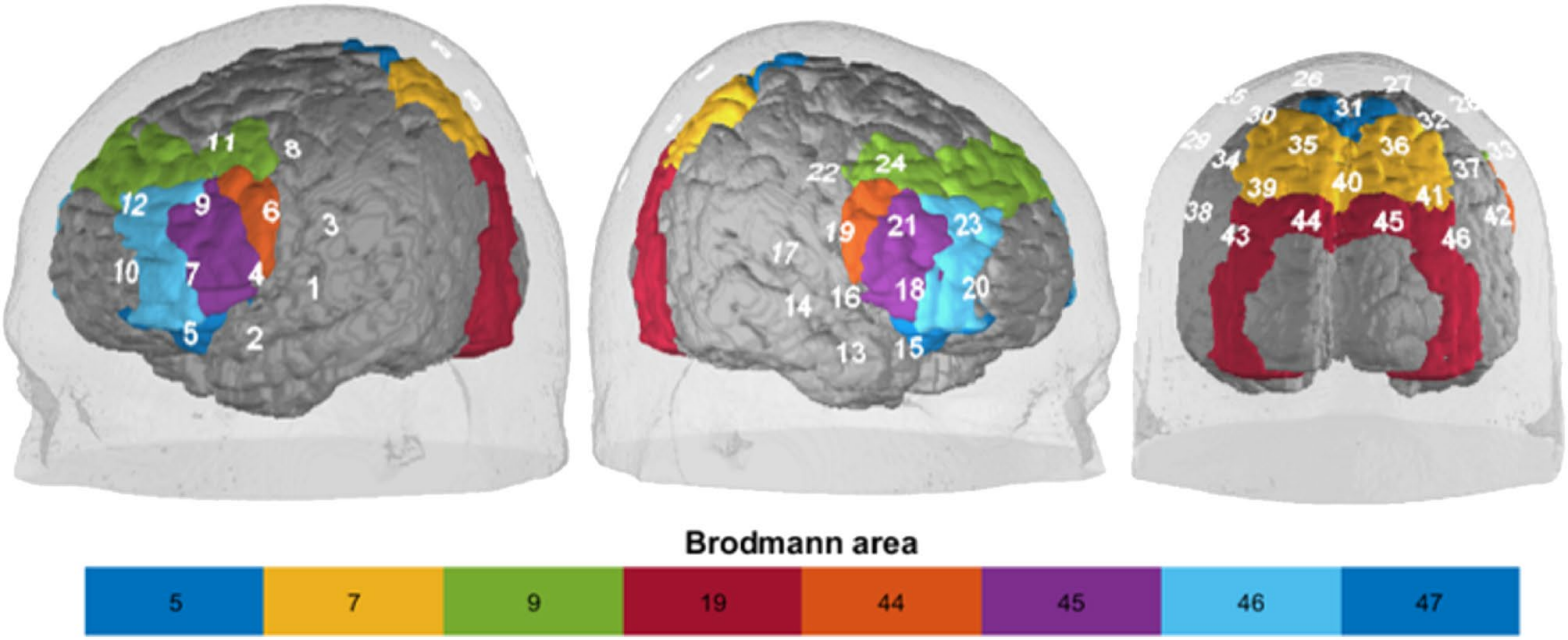
Placement of the three probesets. Numbers represent channels (see Table 2). Please note that the probesets were integrated into EEG-Easycaps with additional sponge rings to ensure optimal placement. Scalp-brain correspondence was estimated based on Okamoto et al.^50^, Okamoto and Dan^51^, as well as Singh et al.^52^.

Using an ETG-4000 Optical Topography System with a sampling rate of 10 Hz, we measured cortical blood oxygenation (46-channel continuous wave multichannel fNIRS; Hitachi Medical Co., Japan). Two frontal probesets (with reference positions F3 and F4) and one parietal probeset (with reference positions Pz, P3 and P4) with a fixed 3 cm inter-optode-distance were placed according to the 10–20 reference points (28 light emitters, i.e. semiconductor lasers and 15 light detectors, i.e. avalanche photodiodes at two wavelengths (695 ± 20 nm and 830 ± 20 nm) with 2.0 ± 0.4 mW for each wavelength at each optode). Relative changes in oxygenated (O2Hb) and deoxygenated (HHb) hemoglobin were computed using self-written MATLAB 2017 scripts by means of a modified Beer-Lambert Law^47^. Preprocessing included the interpolation of single noisy channels, correction of motion artifacts using Temporal Derivative Distribution Repair^48^, Correlation-based signal improvement^49^ and bandpass-filtering to remove low-frequency baseline-drifts (< 0.01 Hz) and high-frequency noise (> 0.1 Hz). In order to remove artifacts due to data correction, another channel interpolation followed and we used a global signal reduction with a spatial gaussian kernel filter (σ = 40). For data analysis, we calculated event-related averages for each trial including a 5 s baseline correction. For a visualization of the probeset placement see Fig. 8. For an assignment of channels to the Regions of Interest (ROI), see Table 2. Lastly, we exported the data for each channel of our ROIs separately: left Inferior Frontal Gyrus (lIFG), right Inferior Frontal Gyrus (rIFG), left Dorsolateral Prefrontal Cortex (lDLPFC), right Dorsolateral Prefrontal Cortex (rDLPFC) and Somatosensory Association Cortex (SAC). Scalp-brain correspondence was estimated based on Okamoto et al.^50^, Okamoto and Dan^51^, as well as Singh et al.^52^.

**Table 2:** Assignment of channels to the defined Regions of Interest.

ROI	Channel
left IFG	7 9 6
left DLPFC	10 12 11
right IFG	18 21 19
right DLPFC	20 23 24
SAC	27 26 25 28 30 31 32 35 36

*IFG* Inferior Frontal Gyrus, *DLPFC* Dorsolateral Prefrontal Cortex, *SAC* Somatosensory Association Cortex.

### Data analysis

**Figure 9:**
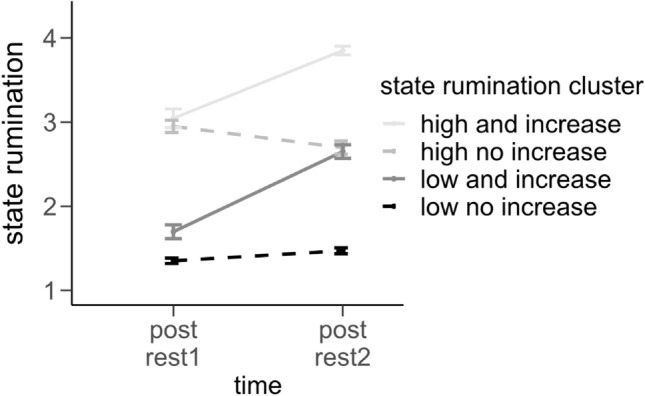
Illustration of state rumination ratings dependent on state rumination clusters. Post rest1 = prior to the stress induction; post rest2 = after the stress induction. Error bars indicate standard errors (± 1 SE).

**Figure 10:**
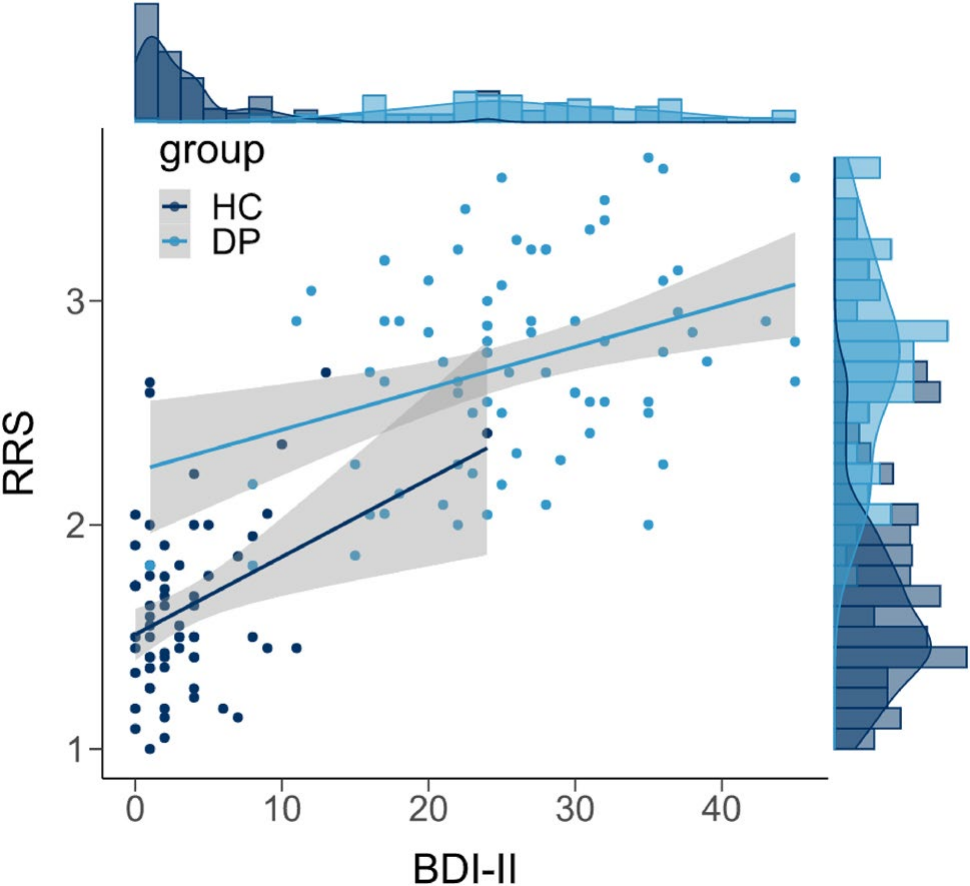
Scatterplot with marginal histograms and density plots of RRS and BDI-II scores dependent on group (*HC* healthy controls, *DP* depressed patients).

Data analysis was done using IBM SPSS Statistics Version 28. Graphics were plotted using RStudio Version 1.4.1717^53^ and R Version 4.0.3^54^ using the packages ggplot2^55^, ggthemes^56^ and ggExtra^57^. As we aimed to investigate the predictive value of state rumination for the hemodynamic changes due to the stress induction and did not only want to consider change scores but also state rumination levels post-stress (we expected more pronounced differences post-stress and we were primarily interested in the neural correlates of stress-induced rumination), we combined both in one variable by using hierarchical clustering. According to the dendrogram we decided for a 4-cluster solution: One cluster of n = 61 participants showing low state rumination levels in general and little to no change in state rumination due to the stress induction (83.1% of the *n* = 61 were HC), one cluster (*n* = 20) including participants with low baseline rumination but increases due to the TSST (15.4% of the *n* = 20 were HC), one cluster (*n* = 35) including high baseline rumination and increases due to the TSST (1.5% of the *n* = 35 were HC) and a forth cluster (*n* = 24) including high baseline rumination but little to no change due to the TSST (0% of the *n* = 35 were HC) (see Fig. 9, Table 3). Next, we investigated the effects of trait rumination (RRS score), depressive symptomatology (BDI-II score) and group (DP vs. HC). Please note, however, that RRS and BDI-II were overall highly correlated (*r*(137) = 0.786, *p* < 0.001; disattenuated correlation using the psych-package *r* = 0.982^58^), and group membership (DP vs. HC) was strongly associated with BDI-II and RRS scores: Performing a median-split resulted in 84.3% HC in the low RRS group, 91.4% DP in the high RRS group, ${\chi (1)}^{2}$ = 80.669, *p* < 0.001, and 90.1% HC in the low BDI-II group and 98.6% DP in the high BDI-II group, ${\chi (1)}^{2}$ = 110.669, *p* < 0.001 (for an illustration see Fig. 10), which is why we abstained from fitting separate models for the aforementioned three variables of interest instead only including diagnostic group (DP vs. HC) as a predictor in our models. We firstly investigated state rumination ratings and therefore fitted a repeated measurements ANOVA (rmANOVA) dependent on time and group (HC vs. DP). Next, we investigated the effect of group and state rumination cluster on the performance measures of the TSST (number of solved items and errors). Lastly, in order to investigate the fNIRS-data, we fitted separate multivariate repeated measurements ANOVAs (rmMANOVAs) including time and group (DP vs. HC) or state rumination cluster (SR-cluster) as a between-subjects factor, respectively. In order to correct fNIRS-data for potential effects of the number of calculations (e.g. higher increases in cortical oxygenation due to an increased recruitment of corresponding brain regions), we computed the ratio of a subject’s given average O2Hb-concentration and the average performance in the corresponding task (CTL1, CTL2 and the arithmetic task of the TSST)^59^. This ratio resulted in the measure “O2Hb per item solved” [(mmol mm)/item] which we investigated with the same rmMANOVA as previously described. Please note that univariate post-hoc tests as well as pairwise comparisons were corrected using the Benjamini–Hochberg procedure whereas due to the complexity of the fNIRS analysis, pairwise comparisons of the main effect of time are to be found in Supplementary Material S2. In case sphericity assumptions were violated, we corrected using Greenhouse Geisser estimates (in all cases, $\varepsilon >$ 0.75).

**Table 3:** Crosstable investigating the distribution of group (*DP* depressed patients, *HC* healthy controls) dependent on state rumination cluster (SR-cluster).

			SR-cluster				Total
			SR-cluster 1 (high and increase)	SR-cluster 2 (high and no increase)	SR-cluster 3 (low and increase)	SR-cluster 4 (low and little to no increase)	
Group	DP	*n*	35	23	10	7	75
		% Within group	46.7%	30.7%	13.3%	9.3%	100%
		% Within SR-cluster	100%	95.8%	50.0%	11.5%	53.6%
		% of total	25.0%	16.4%	7.1%	5.0%	53.6%
	HC	*n*	0	1	10	54	65
		% Within group	0.0%	1.5%	15.4%	83.1%	100%
		% Within SR-cluster	0.0%	4.2%	50.0%	88.5%	46.5%
		% of total	0.0%	0.7%	7.1%	38.6%	46.4%
Total		*n*	35	24	20	61	140
		% Within group	25.0%	17.1%	14.3%	43.6%	100%
		% Within SR-cluster	100%	100%	100%	100%	100%
		% of total	25.0%	17.1%	14.3%	43.6%	100%

We further excluded two participants whose state rumination ratings were identified as multivariate outliers (*p* < 0.01) according to their Mahalanobis distances. In the following, we will report the results of all channels of our ROIs; however, plots of all significant results of all channels except for the left DLPFC are to be found in the Supplementary Material S3.

### Participants

Before merging both study samples, we performed independent samples *t*-tests comparing the demographic variables of the overall samples as well as the subsamples (HC and DP). Concerning the overall samples, we found no significant differences in depressive symptoms as assessed using the BDI-II, *t*(140) =  − 1.643, *p* = 0.103, *d* =  − 0.296, trait rumination levels as assessed using the RRS, *t*(139) =  − 0.789, *p* = 0.432, *d* =  − 0.142, or the sex distribution, ${\chi (1)}^{2}$ = 0.341, *p* = 0.559. However, we did find the sample of study 1 to be on average 4 years younger compared to the sample of study 2, *t*(135.34) =  − 2.908, *p* < 0.01, *d* =  − 0.431. Comparing the HC subsamples, we found no differences concerning age, *t*(63) =  − 0.899, *p* = 0. 372, *d* = − 0.233, RRS, *t*(63) = 1.651, *p* = 0.104, *d* = 0.428, or the percentage of female participants, ${\chi (1)}^{2}$= 0.001, *p* = 0.977. However, we found HC in study 1 to rate their depressive symptoms as significantly lower compared to HC in study 2, *t*(60.43) =  − 2.232, *p* < 0.05, *d* =  − 0.471. Concerning DP, we found patients of study 1 to be comparable regarding their BDI-II, *t*(27.58) =  − 0.915, *p* = 0.368, *d* =  − 0.282, and RRS, *t*(74) =  − 1.645, *p* = 0.104, *d* =  − 0.416, and sex distribution, ${\chi (1)}^{2}$= 0.515, *p* = 0.473, but DP of study 1 were significantly younger than DP of study 2, *t*(67.17) =  − 2.743, *p* < 0.01, *d* =  − 0.282. For means and standard deviations please see Table 4. The final total sample comprised *n* = 77 DP and* n* = 65 HC. 74.65% of the sample was female with a mean age of 29.01 (*SD* = 9.62) years, mean BDI-II of 15.56 (*SD* = 13.30) and mean RRS of 2.23 (*SD* = 0.69) (see Table 4). Not surprisingly, we found BDI-II and RRS to be highly correlated (*r*(142) = 0.784, *p* < 0.001) (see Fig. 9). The diagnoses in the patient sample included recurrent Major Depressive Disorder (MDD) (*n* = 52), first episode MDD (*n* = 21) as well as *n* = 1 patient with an adjustment disorder and *n* = 3 patients with problems related to life management difficulties. All DP were currently in a depressed state according to their BDI-II score (*M* = 25.91, *SD* = 8.82). 51.3% were currently receiving psychotherapy and 41.6% antidepressant medication.

**Table 4:** Demographic data dependent on the subsamples and the merged total sample.

Study	Variable	Group	*M*	*SD*	Test-statistic comparing DP and HC	*p*-value	Cohen’s *d*
1	Age	DP (*n* = 22)	27.14	6.15	*t*(43) = 1.008	0.319	0.301
		HC (*n* = 23)	25.35	5.75			
	BDI-II	DP (*n* = 22)	24.14	11.85	*t*(22.10) = 8.596	< 0.001	2.619
		HC (*n* = 23)	2.13	1.96			
	RRS	DP (*n* = 22)	2.59	0.50	*t*(43) = 6.447	< 0.001	1.923
		HC (*n* = 23)	1.73	0.39			
	% female	DP (*n* = 22)	77.27%		${\chi }^{2}(1)$ = 0.006	0.936	
		HC (*n* = 23)	78.26%				
2	Age	DP (*n* = 55)	32.60	11.12	*t*(93.985) = 2.548	< 0.01	0.510
		HC (*n* = 42)	27.29	9.39			
	BDI-II	DP (*n* = 55)	26.62	7.29	*t*(91.919) = 18.727	< 0.001	3.619
		HC (*n* = 42)	3.95	4.58			
	RRS	DP (*n* = 55)	2.78	0.44	*t*(94) = 14.415	< 0.001	2.966
		HC (*n* = 42)	1.57	0.37			
	% female	DP (*n* = 55)	69.09%		${\chi }^{2}(1)$ = 1.091	0.296	
		HC (*n* = 42)	78.57%				
Total sample	Age	DP (*n* = 77)	31.04	10.22	*t*(139.795) = 2.856	< 0.01	0.473
		HC (*n* = 65)	26.60	8.29			
	BDI-II	DP (*n* = 77)	25.91	8.82	*t*(108.946) = 20.218	< 0.001	3.217
		HC (*n* = 65)	3.31	3.94			
	RRS	DP (*n* = 77)	2.72	0.46	*t*(139) = 15.270	< 0.001	2.580
		HC (*n* = 65)	1.63	0.38			
	% female	DP (*n* = 77)	71.43%		${\chi }^{2}(1)$ = 0.921	0.334	
		HC (*n* = 65)	78.47%				

Study 1^16^, study 2^32^.

*BDI-II* Beck Depression Inventory II^46, 60^, *RRS* Ruminative Response Scale^2^, *DP* depressed patients, *HC* healthy controls.

### Supplementary Information

Supplementary Information.

## Data Availability

The datasets analyzed during the current study are available from the corresponding author on reasonable request.
