# Supplementary material for: Prefrontal hypoactivation induced via social stress is more strongly associated with state rumination than depressive symptomatology
Source: Sci Rep. 2023 Sep 13;13:15147. doi: 10.1038/s41598-023-41403-y (PMC10499935; doi:10.1038/s41598-023-41403-y)
Supplement: Supplementary file 1 — Supplementary Information. [file 41598_2023_41403_MOESM1_ESM.pdf]

Prefrontal hypoactivation induced via social stress is more strongly associated with state rumination than depressive symptomatology

Isabell Int-Veen[1], Andreas J. Fallgatter[2], Ann-Christine Ehlis[2], David Rosenbaum[1]

[1] Department of Psychiatry and Psychotherapy, University Hospital of Tuebingen, Tübingen Center for Mental Health (TüCMH), Tuebingen, Germany
[2] Tübingen Center for Mental Health (TüCMH), Tuebingen, Germany and German Center for Mental Health (DZPG)

Corresponding Author:
Isabell Int-Veen
Calwerstraße 14
72076 Tübingen
Germany
email: isabell.int-veen@med.uni-tuebingen.de

Supplementary material

Supplementary material S1. Items of the state rumination questionnaire including adapted items from the Ruminative Response Scale (Nolen-Hoeksema & Morrow, 1991), Amsterdam Resting-State Questionnaire (Diaz et al., 2013) and the Perseverative Thinking Questionnaire (Ehring et al., 2011). Subjects were instructed to rate if the items were in line with their mental state during the last resting-state measurement.

**German translation**	**English translation**
Ich dachte immer wieder an meine Probleme.	I repeatedly thought about my problems.
Ich verharrte im Denken an Dinge, die mich beunruhigen.	I kept thinking about things that bother me.
Meine Gedanken wiederholten sich, ohne dass ich zu einer Lösung kam.	I dwelled on my thoughts without coming to a solution.
Ich verlor mich in meinen negativen Gedanken.	I got lost in my negative thoughts.
Ich konnte meine Gedanken nur mühsam festhalten.	I had difficulties holding on to my thoughts.
Ich konnte mich nicht von meinen negativen Gedanken lösen.	I could not let go of my negative thoughts.
Ich war bei der Sache.	I was present.
Ich dachte darüber nach, warum ich mich in bestimmten Situationen falsch verhalten habe.	I thought about why I acted wrong in certain situations.
Ich fragte mich, warum ich Probleme habe, die andere nicht haben.	I thought why I have problems other people don´t have.
Ich fragte mich, womit ich meine momentane Lebenssituation verdient habe.	I thought about whereby I deserved my current life situation.
Ich dachte darüber nach, warum ich die Dinge nicht besser in den Griff bekomme.	I thought why I can´t handle things better.
Ich dachte an all meine Defizite und Misserfolge, Macken und Fehler.	I thought about all my shortcomings, failings, faults, mistakes.
Ich konnte flexibel zwischen meinen Gedanken hin und her schalten.	I could switch between my thoughts flexibly.
Ich dachte an vergangene Situationen, die ich bereue.	I thought about past situations that I regret.
Ich machte mir Selbstvorwürfe.	I blamed myself.
Ich verlor mich in Gedanken an Vergangenes.	I got lost in thoughts about the past.
Ich war von meinen Problemen und Sorgen stark vereinnahmt.	I was consumed by my problems and worries.
Meine negativen Gedanken ließen mich nicht los.	I couldn´t let go of my negative thoughts.

Supplementary material S2. Results of Benjamini-Hochberg-corrected pairwise comparisons main effect of time of the NIRS-analysis.

NIRS dependent on group (DP vs. HC): Benjamini-Hochberg-corrected post-hoc pairwise comparisons of the main effect of time (CTL1 vs. TSST and CTL2 vs. TSST for significant channels) indicated significant increases from CTL1 to the TSST in the case of all three channels of the left IFG, left DLPFC, right DLPFC, all nine channels of the SAC as well as one of three channels of the right IFG (channel 18) respectively. Significant increases from CTL2 to TSST were observed in the case of all three channels of the left DLPFC, two out three channels of the left IFG (channel 6, 9), two out of three channels of the right DLPFC (channel 20. 24) and two out of nine channels of the SAC (channel 27, 31).

NIRS dependent on SR-cluster: We investigated the Benjamini-Hochberg-corrected pairwise comparisons of the main effect of time and observed significant increases from CTL1 to the TSST in the case of two channels of the left IFG (channel 7, 9), one channel of the right DLPFC (channel 20), and two channels covering the SAC (channel 25, 32). We further observed significant increases from CTL2 to the TSST in the case of a subset of the aforementioned channels, namely channel 9 and 20.

Performance-corrected NIRS dependent on group (DP vs. HC): Pairwise comparisons of the time effect in the case of these channels yielded significant differences between CTL1 and TSST in the case of all channels of the left and right DLPFC, all channels of the SAC as well as two out of three channels of the left IFG (channel 6, 9). We further observed significant differences between CTL2 and TSST in the case of all channels of the left DLPFC, two out of three channels of the right DLPFC (channel 20. 24), a subset of two channels of the SAC (channel 27, 31) as well as the same channels of the left IFG (channel 6, 9).

Performance-corrected NIRS dependent on SR-cluster: Pairwise comparisons of the time effect in these channels yielded significant increases between CTL1 and TSST in the case of all channels of the right DLPFC, one channel of the left DLPFC (channel 10), seven out of nine channels of the SAC (channel 25, 26, 27, 28, 30. 32. 35) and one channel of the left IFG (channel 6). We further observed significant increases between CTL2 and TSST in the case of three channels of the SAC (25, 28, 32).

Supplementary material S3. Illustration of the results of the fNIRS-MANOVAs. Please note that only channels with significant group*time interactions are depicted. Error bars indicate +/- 1 SE.

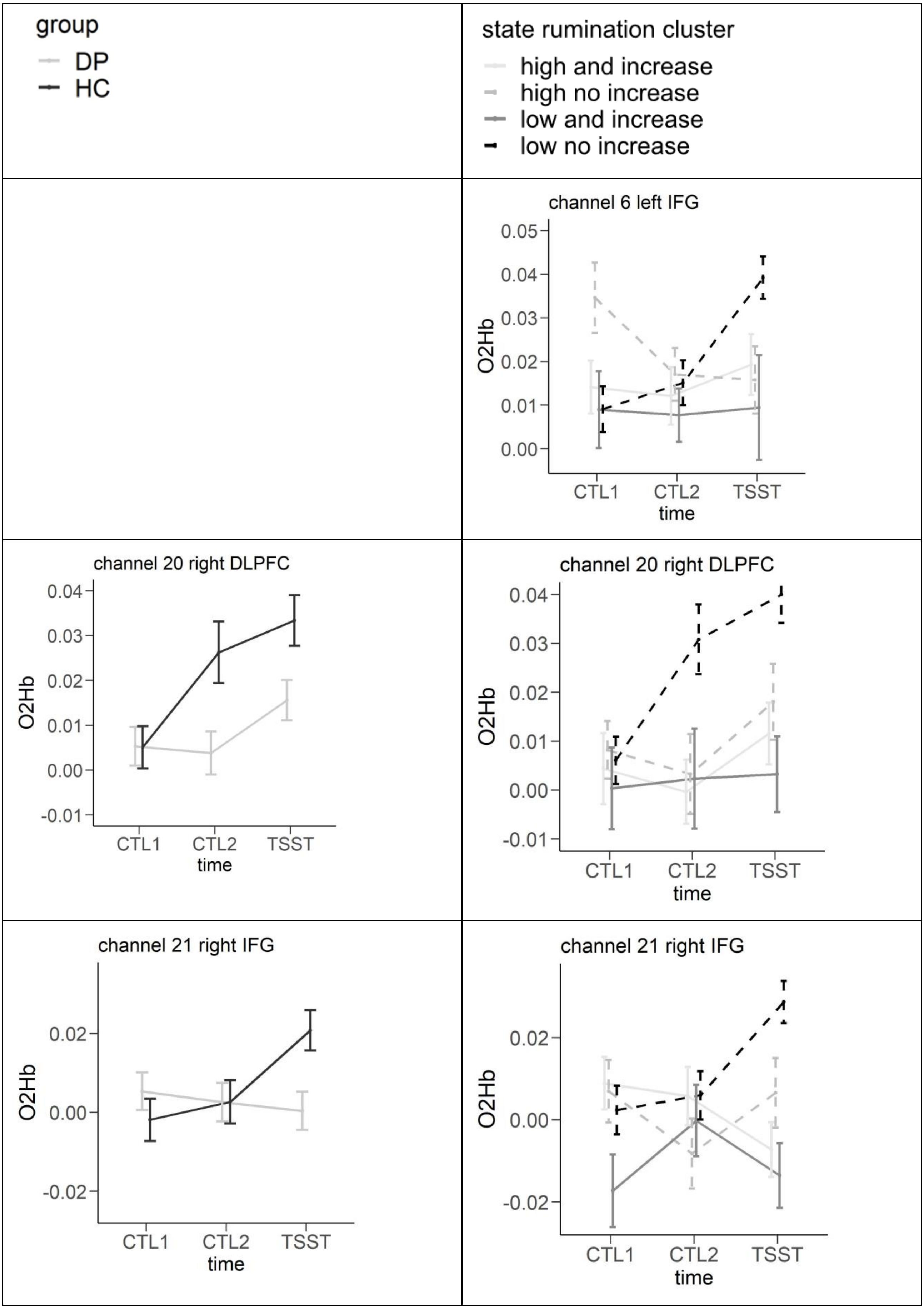

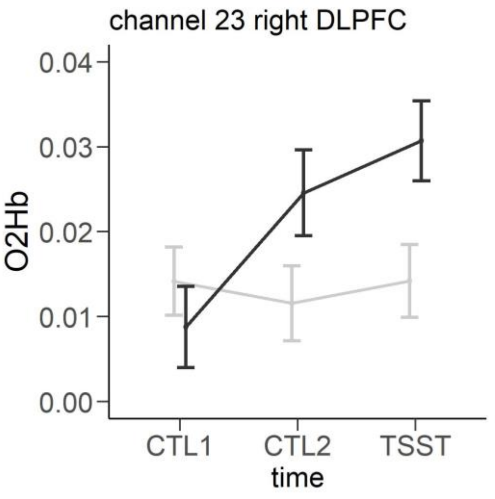

channel 23 right DLPFC
O2Hb
0.04
0.03
0.02
0.01
0.00
CTL1
CTL2
TSST
time

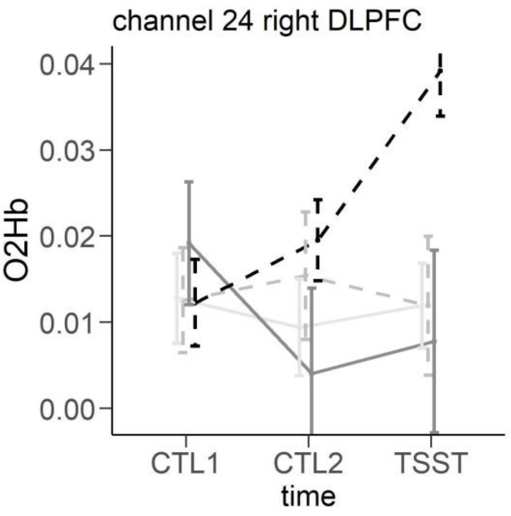

channel 24 right DLPFC
O2Hb
0.04
0.03
0.02
0.01
0.00
CTL1
CTL2
TSST
time

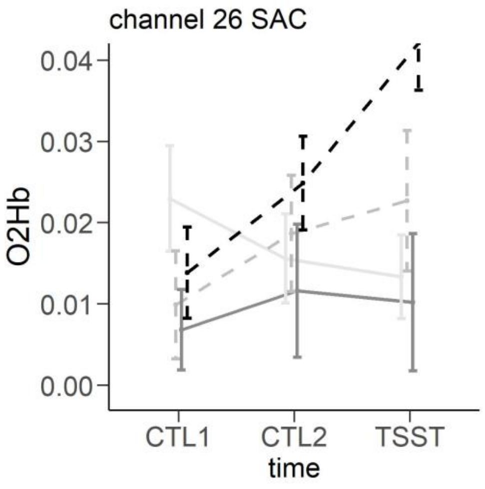

channel 26 SAC
O2Hb
0.04
0.03
0.02
0.01
0.00
CTL1
CTL2
TSST
time

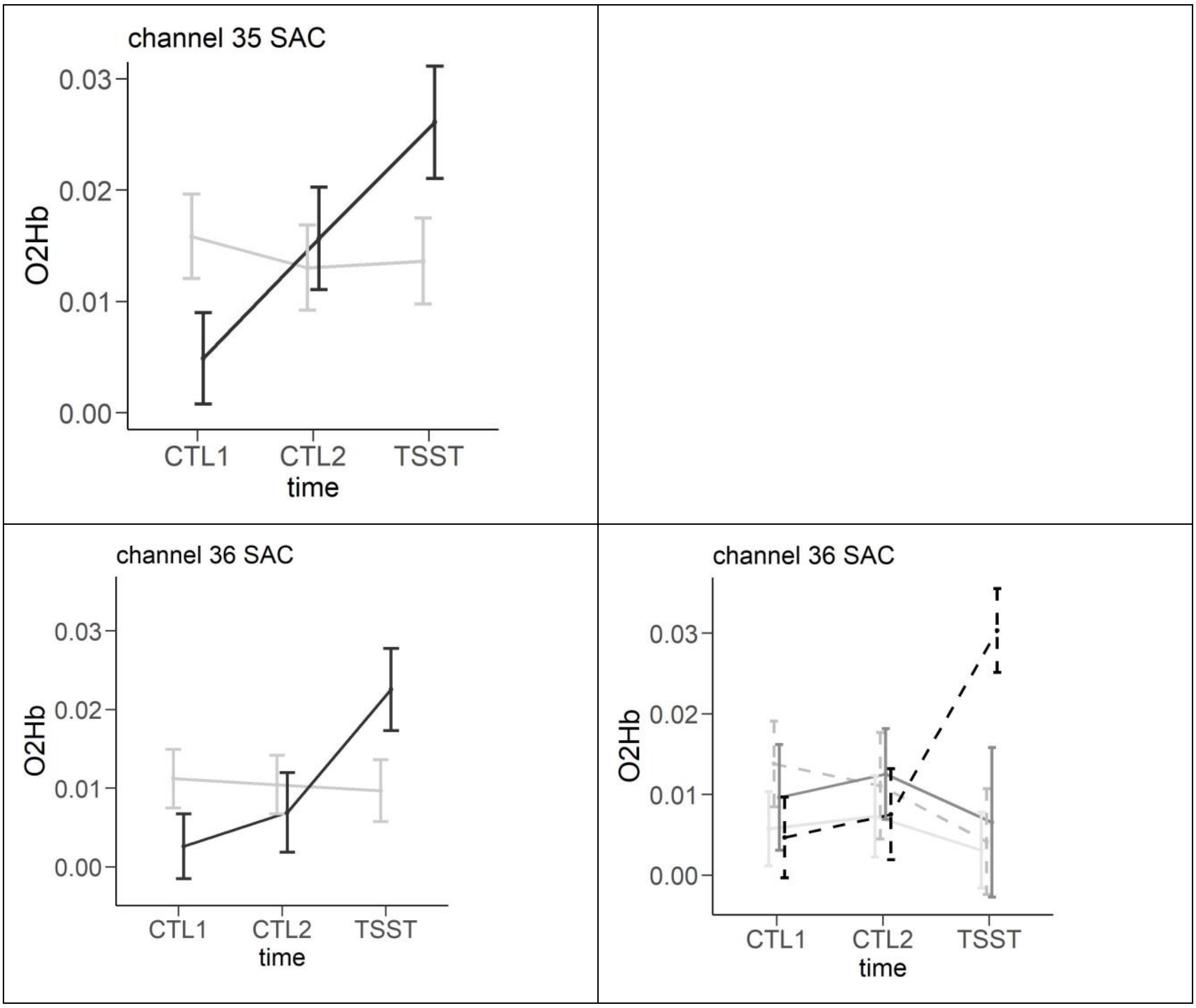
channel 35 SAC
O2Hb
0.03
0.02
0.01
0.00
CTL1
CTL2
TSST
time
channel 36 SAC
O2Hb
0.03
0.02
0.01
0.00
CTL1
CTL2
TSST
time
channel 36 SAC
O2Hb
0.03
0.02
0.01
0.00
CTL1
CTL2
TSST
time

Supplementary material S4. T-tests investigating the differences in NIRS cortical oxygenation dependent on group (DP = depressed patients vs. HC = healthy controls). Please note that this is for illustrative purposes only and therefore not corrected for multiple comparisons. Note: p-value of one-sided t-tests

	group	N	M	SD	t	df	*p*	95% CI	effect size *d*
CTL1 Ch6	HC	65	0.008	0.040	-1.853	138	0.033	[-0.026; 0.001]	-0.314
	DP	75	0.020	0.039					
	total sample	140	0.015	0.040					
CTL1 Ch7	HC	65	-0.006	0.034	0.540	138	0.295	[-0.008; 0.015]	0.092
	DP	75	-0.009	0.035					
	total sample	140	-0.008	0.034					
CTL1 Ch9	HC	65	-0.008	0.037	-1.441	124.59	0.076	[-0.020; 0.003]	-0.248
	DP	75	0.000	0.030					
	total sample	140	-0.003	0.033					
CTL1 Ch10	HC	65	0.006	0.033	-0.365	138	0.358	[-0.013; 0.009]	-0.062
	DP	75	0.008	0.033					
	total sample	140	0.007	0.033					
CTL1 Ch11	HC	65	0.002	0.038	-1.986	138	0.024	[-0.024; 0.000]	-0.337
	DP	75	0.013	0.032					
	total sample	140	0.008	0.036					
CTL1 Ch12	HC	65	0.009	0.036	-0.480	138	0.316	[-0.015; 0.009]	-0.081
	DP	75	0.012	0.036					
	total sample	140	0.010	0.036					
CTL1 Ch18	HC	65	-0.009	0.029	0.123	138	0.451	[-0.011; 0.012]	0.021
	DP	75	-0.010	0.038					
	total sample	140	-0.010	0.034					
CTL1 Ch19	HC	65	0.018	0.038	0.033	138	0.487	[-0.014; 0.014]	0.006
	DP	75	0.018	0.046					
	total sample	140	0.018	0.042					
CTL1 Ch20	HC	65	0.005	0.038	-0.030	138	0.488	[-0.013; 0.012]	-0.005
	DP	75	0.005	0.037					
	total sample	140	0.005	0.037					
CTL1 Ch21	HC	65	-0.002	0.043	-1.008	138	0.158	[-0.021; 0.007]	-0.171

	DP	75	0.005	0.041					
	total sample	140	0.002	0.042					
CTL1 Ch23	HC	65	0.009	0.039	-0.869	138	0.193	[-0.018; 0.007]	-0.147
	DP	75	0.014	0.035					
	total sample	140	0.012	0.037					
CTL1 Ch24	HC	65	0.010	0.033	-0.963	138	0.169	[-0.017; 0.006]	-0.163
	DP	75	0.016	0.036					
	total sample	140	0.013	0.035					
CTL1 Ch25	HC	65	0.012	0.037	-1.472	138	0.072	[-0.022; 0.003]	-0.249
	DP	75	0.021	0.037					
	total sample	140	0.017	0.037					
CTL1 Ch26	HC	65	0.010	0.036	-1.295	138	0.099	[-0.021; 0.004]	-0.219
	DP	75	0.018	0.040					
	total sample	140	0.014	0.038					
CTL1 Ch27	HC	65	0.011	0.033	-0.550	138	0.292	[-0.015; 0.008]	-0.093
	DP	75	0.014	0.036					
	total sample	140	0.013	0.034					
CTL1 Ch28	HC	65	0.014	0.038	-1.215	138	0.113	[-0.020; 0.005]	-0.206
	DP	75	0.022	0.036					
	total sample	140	0.018	0.037					
CTL1 Ch30	HC	65	0.011	0.043	-1.854	138	0.033	[-0.027; 0.001]	-0.314
	DP	75	0.024	0.040					
	total sample	140	0.018	0.041					
CTL1 Ch31	HC	65	0.004	0.035	-1.583	138	0.058	[-0.020; 0.002]	-0.268
	DP	75	0.013	0.031					
	total sample	140	0.009	0.033					
CTL1 Ch32	HC	65	0.008	0.034	-1.721	138	0.044	[-0.022; 0.002]	-0.292
	DP	75	0.018	0.037					
	total sample	140	0.014	0.036					
CTL1 Ch35	HC	65	0.005	0.033	-1.956	138	0.026	[-0.022; 0.000]	-0.332
	DP	75	0.016	0.033					
	total sample	140	0.011	0.033					
CTL1 Ch36	HC	65	0.003	0.033	-1.550	138	0.062	[-0.020; 0.002]	-0.263

	DP	75	0.011	0.032					
	total sample	140	0.007	0.033					
CTL2 Ch6	HC	65	0.014	0.041	0.004	138	0.498	[-0.012; 0.012]	0.001
	DP	75	0.014	0.033					
	total sample	140	0.014	0.036					
CTL2 Ch7	HC	65	0.011	0.045	1.045	138	0.149	[-0.007; 0.022]	0.177
	DP	75	0.003	0.040					
	total sample	140	0.007	0.042					
CTL2 Ch9	HC	65	-0.007	0.045	-0.542	138	0.294	[-0.018; 0.010]	-0.092
	DP	75	-0.003	0.039					
	total sample	140	-0.005	0.041					
CTL2 Ch10	HC	65	0.019	0.054	1.524	138	0.065	[-0.004; 0.028]	0.258
	DP	75	0.007	0.042					
	total sample	140	0.012	0.048					
CTL2 Ch11	HC	65	0.004	0.037	-0.862	138	0.195	[-0.019; 0.007]	-0.146
	DP	75	0.010	0.040					
	total sample	140	0.007	0.039					
CTL2 Ch12	HC	65	0.009	0.047	-0.151	138	0.440	[-0.015; 0.013]	-0.026
	DP	75	0.010	0.039					
	total sample	140	0.010	0.043					
CTL2 Ch18	HC	65	0.006	0.045	1.835	138	0.034	[-0.001; 0.030]	0.311
	DP	75	-0.008	0.047					
	total sample	140	-0.002	0.046					
CTL2 Ch19	HC	65	0.023	0.041	1.715	138	0.044	[-0.002; 0.025]	0.291
	DP	75	0.011	0.039					
	total sample	140	0.017	0.040					
CTL2 Ch20	HC	65	0.026	0.056	2.723	138	0.004	[0.006; 0.039]	0.462
	DP	75	0.004	0.042					
	total sample	140	0.014	0.050					
CTL2 Ch21	HC	65	0.003	0.044	0.013	138	0.495	[-0.014; 0.015]	0.002
	DP	75	0.003	0.042					
	total sample	140	0.003	0.043					
CTL2 Ch23	HC	65	0.025	0.041	1.945	138	0.027	[0.000; 0.026]	0.330

	DP	75	0.012	0.038					
	total sample	140	0.018	0.040					
CTL2 Ch24	HC	65	0.014	0.037	-0.125	138	0.450	[-0.013; 0.012]	-0.021
	DP	75	0.014	0.037					
	total sample	140	0.014	0.037					
CTL2 Ch25	HC	65	0.030	0.044	-0.358	138	0.361	[-0.017; 0.012]	-0.061
	DP	75	0.033	0.040					
	total sample	140	0.032	0.042					
CTL2 Ch26	HC	65	0.021	0.040	0.307	138	0.380	[-0.011; 0.015]	0.052
	DP	75	0.019	0.039					
	total sample	140	0.020	0.039					
CTL2 Ch27	HC	65	0.014	0.037	-1.165	138	0.123	[-0.020; 0.005]	-0.197
	DP	75	0.022	0.038					
	total sample	140	0.018	0.037					
CTL2 Ch28	HC	65	0.028	0.040	-0.841	138	0.201	[-0.019; 0.008]	-0.142
	DP	75	0.034	0.041					
	total sample	140	0.032	0.041					
CTL2 Ch30	HC	65	0.030	0.046	0.815	138	0.208	[-0.009; 0.021]	0.138
	DP	75	0.024	0.042					
	total sample	140	0.027	0.044					
CTL2 Ch31	HC	65	0.012	0.047	1.135	110.74	0.129	[-0.006; 0.021]	0.197
	DP	75	0.004	0.032					
	total sample	140	0.008	0.039					
CTL2 Ch32	HC	65	0.024	0.048	-0.779	138	0.219	[-0.020; 0.009]	-0.132
	DP	75	0.030	0.038					
	total sample	140	0.027	0.043					
CTL2 Ch35	HC	65	0.016	0.037	0.443	138	0.329	[-0.009; 0.014]	0.075
	DP	75	0.013	0.033					
	total sample	140	0.014	0.035					
CTL2 Ch36	HC	65	0.007	0.041	-0.564	120.92	0.287	[-0.016; 0.009]	-0.097
	DP	75	0.010	0.032					
	total sample	140	0.009	0.036					
TSST Ch6	HC	65	0.029	0.039	0.789	138	0.216	[-0.009; 0.020]	0.134

	DP	75	0.023	0.046					
	total sample	140	0.026	0.043					
TSST Ch7	HC	65	0.013	0.046	-0.358	138	0.361	[-0.023; 0.016]	-0.061
	DP	75	0.017	0.066					
	total sample	140	0.015	0.058					
TSST Ch9	HC	65	0.013	0.035	0.763	138	0.223	[-0.008; 0.018]	0.129
	DP	75	0.008	0.042					
	total sample	140	0.011	0.039					
TSST Ch10	HC	65	0.030	0.039	2.595	138	0.005	[0.004; 0.029]	0.440
	DP	75	0.014	0.036					
	total sample	140	0.021	0.038					
TSST Ch11	HC	65	0.023	0.036	1.744	138	0.042	[-0.002; 0.024]	0.296
	DP	75	0.012	0.039					
	total sample	140	0.017	0.038					
TSST Ch12	HC	65	0.031	0.040	2.605	138	0.005	[0.004; 0.030]	0.442
	DP	75	0.013	0.038					
	total sample	140	0.021	0.040					
TSST Ch18	HC	65	0.009	0.045	1.394	138	0.083	[-0.004; 0.024]	0.236
	DP	75	-0.001	0.040					
	total sample	140	0.003	0.042					
TSST Ch19	HC	65	0.029	0.036	1.699	138	0.046	[-0.002; 0.024]	0.288
	DP	75	0.018	0.041					
	total sample	140	0.023	0.039					
TSST Ch20	HC	65	0.033	0.045	2.492	138	0.007	[0.004; 0.032]	0.422
	DP	75	0.016	0.039					
	total sample	140	0.024	0.043					
TSST Ch21	HC	65	0.021	0.041	2.878	138	0.002	[0.006; 0.034]	0.488
	DP	75	0.000	0.042					
	total sample	140	0.010	0.043					
TSST Ch23	HC	65	0.031	0.038	2.593	138	0.005	[0.004; 0.029]	0.439
	DP	75	0.014	0.037					
	total sample	140	0.022	0.038					
TSST	HC	65	0.030	0.042	1.879	138	0.031	[-0.001; 0.027]	0.318

Ch24	DP	75	0.017	0.040					
	total sample	140	0.023	0.041					
TSST Ch25	HC	65	0.032	0.037	0.482	138	0.315	[-0.009; 0.015]	0.082
	DP	75	0.029	0.036					
	total sample	140	0.030	0.037					
TSST Ch26	HC	65	0.034	0.043	1.712	138	0.045	[-0.002; 0.026]	0.290
	DP	75	0.021	0.041					
	total sample	140	0.027	0.042					
TSST Ch27	HC	65	0.030	0.039	1.270	138	0.103	[-0.005; 0.021]	0.215
	DP	75	0.022	0.037					
	total sample	140	0.025	0.038					
TSST Ch28	HC	65	0.031	0.040	0.551	138	0.291	[-0.010; 0.017]	0.093
	DP	75	0.028	0.041					
	total sample	140	0.029	0.041					
TSST Ch30	HC	65	0.034	0.043	0.840	138	0.201	[-0.008; 0.020]	0.0142
	DP	75	0.028	0.040					
	total sample	140	0.030	0.042					
TSST Ch31	HC	65	0.025	0.045	1.793	138	0.038	[-0.001; 0.027]	0.304
	DP	75	0.012	0.039					
	total sample	140	0.018	0.042					
TSST Ch32	HC	65	0.034	0.053	0.895	138	0.186	[-0.009; 0.023]	0.152
	DP	75	0.027	0.040					
	total sample	140	0.030	0.046					
TSST Ch35	HC	65	0.026	0.041	1.994	138	0.024	[0.000; 0.025]	0.338
	DP	75	0.014	0.033					
	total sample	140	0.019	0.037					
TSST Ch36	HC	65	0.023	0.042	1.997	138	0.024	[0.000; 0.026]	0.338
	DP	75	0.010	0.034					
	total sample	140	0.016	0.038					
